# Headache providers' perspectives of headache diaries in the era of increasing technology use: a qualitative study

**DOI:** 10.3389/fneur.2023.1270555

**Published:** 2024-01-23

**Authors:** Mia Minen, Alexis George, Naomi Lebowitz, Aarti Katara, Ivy Snyder

**Affiliations:** ^1^Department of Neurology, NYU Langone Health, New York, NY, United States; ^2^Barnard College, New York, NY, United States; ^3^Ferkauf Graduate School of Psychology, New York, NY, United States; ^4^Department of Psychology, Yeshiva University, New York, NY, United States

**Keywords:** headache, migraine, provider perspective, headache diary, symptom tracking, digital health

## Abstract

**Background:**

No matter what type of headache is being considered across various populations, one of the mainstays of headache medicine is headache tracking. This self-management tool enables patients and their providers to understand patients' underlying symptoms and the effects of treatments they have tried. This is important to determining whether headaches are related to menses for women's health, to determining the time of headache occurrence, e.g., hypnic headache, and the location and duration of symptoms, e.g., trigeminal autonomic cephalgia. Prior research has investigated what people with headaches perceive about headache diary use and how people with headaches utilize electronic headache diaries. However, headache providers' perspectives on the important factors related to headache diaries are less known. Previously, using the Modified Delphi Process, a panel of four experts opined what they perceived as the most important factors for a headache diary. We sought to better understand headache providers' perspectives about headache diary/app usage from providers working in various institutions nationwide.

**Methods:**

We conducted 20 semi-structured qualitative interviews of headache providers across the US from various institutions and asked them their perspectives on headache diary use. We transcribed the interviews, which two independent coders then coded. Themes and subthemes were developed using grounded theory qualitative analysis.

**Results:**

Six themes emerged: (1) Providers were generally agnostic regarding the headache tracking method, but nearly all recommend the use of smartphones for tracking; (2) Providers had concerns regarding the accessibility of headache trackers; (3) Providers noted benefits to integrating headache tracking data into the EMR but had mixed opinions on how this integration might be done; (4) Providers had mixed opinions regarding the utility and interpretation of the data, specifically regarding data accuracy and efficiency; (5) Providers generally felt that headache tracking lends itself to more collaborative plan management; (6) Providers recommend behavioral health apps for patients but stated that there are few digital behavioral health interventions for headache specifically.

**Conclusion:**

Interviews of headache providers, recommenders, and users of headache data are vital informants who can provide a robust amount of information about headache diary development, use in different populations, integration, and more.

## Introduction

Maintaining a headache diary to track headaches' frequency, characteristics, and severity is crucial to headache and migraine care (1–3). Digital headache tracking using a smartphone is readily available to patients: patients 18–50 are most likely to present for migraine care, and 89–94% of people in this age range in the US have smartphone access (4). Increasingly, patients are moving toward digitally based solutions for their migraine management; many popular, commercially available headache and migraine-tracking smartphone applications (apps) for Android and iOS systems exist (3–5). However, few apps have been developed with physician input (6, 7). Our team has studied the feasibility, acceptability, satisfaction, and efficacy of a headache-tracking app developed with patient and provider user input (4–6, 8–12).

Our prior research has investigated what people with headache perceive about headache diary use and how people with headache utilize electronic headache diaries (5, 8). We have examined the following questions: (1) what are headache smartphone app users looking for in apps (5)? We investigated this through the analysis of user reviews of commercially available electronic headache diary apps, and (2) what do people with migraine consider to be essential features of migraine tracking (8)?

In addition to headache patient perspectives, our team has studied the feasibility, acceptability, satisfaction, and efficacy of a headache-tracking app, RELAXaHEAD, developed with patient and provider user input (4–6, 8–12). We assessed qualitative diary usage across our RELAXaHEAD clinical trials. RELAXaHEAD clinical trials have been conducted to assess practicality and appropriateness for patients with migraine in primary care, the emergency department, urgent care, those who have posttraumatic headache, and patients with multiple sclerosis (4–6, 8–12). Thus, we have developed a rich understanding of patient priorities regarding electronic headache diaries. However, in this evolving market, little is known about headache providers' perspectives on headache diaries.

Previously, using the Modified Delphi Process, a panel of four experts opined on what they perceived as the most important factors for a headache diary (1). They identified what they perceived as the most important concepts, “The 3 Fs:” “Frequency of days with headache; Frequency of acute medication usage; and Functional impairment (1).” They also agreed that two separate reports should be generated, one for the providers and one for the patients (1). Several additional features are important in headache tracking. By conducting semi-structured interviews to assess the attitudes and beliefs of providers who treat migraine toward prescribing digital headache smartphone applications and/or electronic headache diaries, we aimed to understand providers' priorities on these topics better to create an informed, data-driven path forward for the implementation of previously developed electronic headache diaries to enhance the treatment and care plans of patients and streamline providers' workflow.

## Methods

We developed and iteratively refined a semi-structured interview guide consisting of 20 questions. The guide was developed by a diverse team of technology experts, including outside experts from the Center for Advancing Point of Care Technologies (CAPCaT) at the University of Massachusetts, a telehealth expert who consults for the American Academy of Neurology, and experts in digital psychiatry at outside institutions. This was to ensure the appropriateness of the content, that the wording captured the intended message, and that bias was minimized. The same interview guide was used throughout the study. Interviews were conducted with 20 headache providers via WebEx and were audio recorded for qualitative analysis. This study was conducted virtually from the New York University (NYU) Grossman School of Medicine (NYUGSoM), located in New York City, with the approval of the NYU Langone Health Institutional Review Board. Informed e-consent was obtained before beginning the interview.

### Recruitment

Eligible participants for this study included headache providers with an MD, DO, NP, or PA degree who treated patients with migraine and actively worked with patients with migraine for at least one full day or the equivalent of a week. Potential participants were emailed information about the study using an NYUGSoM IRB-approved script and an attached information sheet. Emails were sent via the NIH Pain Consortium Network, the American Headache Society Primary Front Line Headache Care Special Interest Section listserv, and the social media platforms Twitter and Facebook. Potential participants were informed that they would receive a $200 check to complete an hour-long semi-structured interview. Interested individuals reached out to the study coordinator via email to schedule a semi-structured interview. An email with instructions for the semi-structured interview and a link to the WebEx video visit was sent to scheduled individuals.

### Enrollment and interview

Audio and informed e-consent were collected from the participants via REDCap software by the study coordinator. Participants were asked demographic questions, whether they had completed a headache medicine fellowship, years in clinical practice, type of institution, and experience with telehealth (Table 1). A health psychology Ph.D. student conducted semi-structured interviews with 20 headache providers using an IRB-approved interview guide (Table A1). The interviews were audio recorded, fully transcribed, and coded using general thematic analysis in Microsoft Word as described below. As the interviews focused on headache providers' perspectives on headache diaries and remote monitoring, we describe their perspectives regarding headache diaries in this paper.

**Table 1:** Clinician demographics.

**Variable**	**Characteristics**	**Frequency (*N* = 20)**	**Percent**
Gender	Male	9	45%
	Female	11	55%
Ethnicity	Non-Hispanic/Latino	17	85%
	Hispanic/Latino	3	15%
Racial group	Asian or Pacific Islander	2	10%
	White/Caucasian	14	70%
	Other	4	20%
Fellowship area of study	Cognitive Neurology	1	5%
	Headache	12	60%
	Did not complete a fellowship	7	35%
Number of years in clinical practice	1–5	6	30%
	6–10	5	25%
	11–20	3	15%
	21–25	1	5%
	>25	2	10%
Institution type	Large Academic	9	45%
	Small Academic	3	15%
	Large Private	2	10%
	Small Private	6	30%

### Analysis

Any personally identifying information (PII) was removed from the transcripts, and qualitative analysis of provider interviews was conducted iteratively by four different coders, MTM (clinician-headache researcher with significant training and experience in qualitative analysis), AG, the research coordinator who works on headache diary research, and two undergraduate students trained in the qualitative methods. The transcripts were divided into two distinct groups based on the interview guide. Group 1 consisted of questions related to headache diaries, and Group 2 consisted of questions specific to remote monitoring. Once the transcripts had been separated, they were divided so that the four independent coders coded the interviews separately. MTM and another independent coder iteratively coded the first three interview transcripts. Then there was a discussion amongst all the coders to ensure consistency in the coding process and to ensure the coders were on track to uncover the nuances in the transcripts. Codes were generated from interview content consistent with grounded theory practices (13). We utilized grounded theory to assess how participants formed significance from our interview questions and as a qualitative coding method that centers on constructing conceptual outlines through building inductive analysis (13). All codes were aligned via mutual agreement by coders. After the interview transcripts had been independently coded, the study coordinator integrated and listed the codes by transcript.

## Results

### Quantitative results

Participants were 45% (9/20) male and 55% (11/20) female. The majority (65%, 13/20) had completed a fellowship. The mean number of years of clinical practice was 11.7, with IQR = 8.75 (Table 1). Interviews lasted an average of 38 ± 0.34 (26–51) min. They covered a range of topics as shown in Table A1.

All 20 providers (100%) asked patients to complete headache diaries, and 95% (19/20) reported requesting that all patients track their headache symptoms in some form of a diary. Nearly all providers, 85% (17/20), recommended patients track their headaches using their mobile phones across various means, including in-house apps, free apps, or non-headache-specific software (i.e., their phone's calendar or word processing app). Most providers (65%, 13/20) cited goals around diary completion or diary adherence and reported leaving final tracking methods up to what would be the easiest or most accessible to the patient. Providers also noted recommending apps only to those patients who appeared sufficiently technologically capable of navigating apps. When probed on how providers assessed for capability, some indicated that they assessed based on the age of their patient. Specifically, if the patient was younger, they assumed they were more tech-savvy. Other providers asked the patient if they preferred digital tracking over paper tracking. A minority of providers indicated concerns that patients might become overly fixated on, or discouraged by, their level of disability, as reported in headache tracking.

Participants provided context regarding the most important outcomes of patients' headache diary tracking. Generally, providers reported hoping to receive information on the frequency and severity of patients' headaches, what acute medications patients took, the efficacy of that medication on the attack, and trend tracking, especially around the monthly occurrence and potential menstrual migraine. Some providers (35%, 7/20) reported that they felt patient recall during office visits around headache occurrence was limited and that diaries significantly improved their insight into the reality of patients' headache patterns. Providers reported that objective data from diary completion is used in appointments to evaluate patients' headache trends, assess treatment efficacy and utilization, and subsequently inform treatment changes.

Providers were also asked whether they recommended applications containing behavioral interventions for headache. Most (80%, 16/20) recommended apps for behavioral interventions relevant to headache, but that were non-headache-specific applications, including Calm, Headspace, and Sleepio. Only one provider recommended a headache-specific application, which was proprietary to their institution, while another suggested a diary application containing some interventional guidance (MigraineManager); another recommended an online web course specific to headache. Just 20% of (4/20) providers reported needing to be made aware of any relevant applications to recommend, and just 10% (2/20) had ever recommended a digital program or application intended expressly for headache beyond a headache-specific diary.

### Qualitative results

From the 20 interviews conducted, six key themes emerged: (1) Providers were generally agnostic regarding the headache tracking method, but nearly all recommend the use of smartphones for tracking to at least some patients; (2) Providers had concerns regarding the accessibility of headache trackers; (3) Providers noted benefits to integrating headache tracking data into the EMR but had mixed opinions on how this integration might be done; (4) Providers had mixed opinions regarding the utility and interpretation of the data, specifically regarding data accuracy and efficiency; (5) Providers generally felt that headache tracking lends itself to more collaborative plan management; (6) Providers recommend behavioral health apps for patients but stated that there are few digital behavioral health interventions for headache specifically (Supplementary Table 1). While responses regarding the impact of electronic vs. paper diary use—and diary use, in general—on patient care varied, there was a consistent desire among providers for headache diary use or tracking to be well-suited to the lifestyle of the patient while meeting the basic informational needs of providers along the continuum of headache care.

### Providers were generally agnostic regarding the headache tracking method, but nearly all recommend using smartphones for tracking at least some patients

Most providers who reported requesting headache tracking from patients were agnostic regarding the tracking method. However, nearly all recommend using mobile phones in headache tracking for at least some patients, depending on their apparent tech-savviness and engagement in their care. Many providers reported being concerned that patients would not adhere to difficult diary tracking and that this concern prevented them from recommending methods or applications they felt were too complex; some navigated this concern by asking patients to record directly into a note app or an existing phone calendar. Depending on the severity and frequency of their patients' headaches, clinicians described several tracking methods ranging from simply listing a color (red, yellow, or green) corresponding to the severity of the headache or day to recording potential foods, weather, or stressors that may have triggered a particular headache. Thus, wide variation exists in the method and medium (paper vs. electronic/app diary) of headache tracking clinicians recommend.

### Providers had concerns regarding the accessibility of headache trackers

Additional considerations for headache tracking, particularly electronic/app tracking, emerged regarding affordability, technological accessibility, language, and headache-related disability accommodations. Providers emphasized the need for headache diary apps to be user-friendly, especially for older patients who may be less familiar with smartphone apps but express interest in the technology. The cost of apps was also of concern, with one clinician noting that their patients who rely on Medicaid are unlikely to purchase an app for headache tracking or behavioral intervention. To maximize the number of patients served, apps must also be available in patients' preferred languages. Finally, providers expressed concern that electronic diaries may be particularly challenging to use for patients who experience light sensitivity.

### Providers noted benefits to integrating headache tracking data into the EMR but had mixed opinions on how this integration might be done

Many providers expressed interest in integrating patients' headache tracking with the EMR, and some reported that they typically ask patients to upload PDFs or photos of their diaries (whether paper or electronic) to their clinic's patient portals. From there, providers may manually input the data sent in by patients into their notes in the EMR for a particular office visit. However, providers differed in their desire to manually input this data into the EMR or sync it automatically from a smartphone app the patient may use. Some providers reported experience with apps that directly interfaced with the EMR—to mixed benefit. Ultimately, providers' primary motivations for integrating patients' headache diaries with the EMR were, for patients who use paper diaries, to reduce the patient burden of remembering to bring their tracked data to office visits and to streamline documentation during visits and preview a patient's most recent data in preparation for a visit.

### Providers had mixed opinions regarding the utility and interpretation of the data, specifically regarding data accuracy and efficiency

Providers often remarked that headache diaries are only helpful if they accurately reflect the patient's symptoms/condition. Since patients' data is viable, clinicians reported consulting diary information when developing and assessing treatment plans. This can increase providers' efficiency by minimizing time spent collecting patients' recalled history during visits. In terms of utility, providers cited headache frequency, severity, and medication use as the most important factors for patients to track. Indeed, some providers reported that they only care to ask for these concrete numbers from patients, leaving additional factors like triggers and patterns for the patient to analyze if they are interested.

Similarly, a minority of clinicians interviewed find reviewing diary data during visits unproductive and instead prefer to focus on discussing treatment and/or patient education. Alternatively, other providers were more interested in the nuances of their patients' headache patterns and emphasized the utility of tracking for investigating menstrual migraine. Additionally, providers likely to suggest behavioral or lifestyle modifications to patients often reported seeing patients engage in more intensive tracking of stressors and other headache triggers. Data readability, particularly regarding electronic/app diaries, was essential to providers, as many expressed the desire for automatically generated graphs to illustrate trends and the ability to view data within 1-month, 3-month, and 1-year time frames.

### Providers generally felt that headache tracking lends itself to more collaborative plan management

Overall, clinicians speculated that incorporating data from patients' headache diaries into discussions during office visits would increase patient satisfaction by demonstrating greater collaboration between patients and providers and validating the efforts of patients to engage with their care plan. Providers also emphasized the benefits to patients of identifying headache triggers and patterns using a diary, increasing patients' self-efficacy, and understanding of their condition. A minority of clinicians expressed concern. However, that detailed, intensive tracking may cause some patients to become overly fixated on their condition. This was of particular concern for patients with psychiatric comorbidities.

### Providers recommend behavioral health apps for patients but stated that there are few digital behavioral health interventions for headache specifically

Per participants in this study, the current digital landscape of behavioral health interventions for headaches is minimal. Providers reported knowing few headache-specific services unless they were proprietary to their setting or had been previously. Many providers reported recommending behavioral health applications broadly to patients or recommending patients look up possible interventions on their own. Many reported this was due to a lack of knowledge of, and personal familiarity with, the behavioral health applications available to them. As above, they mentioned offering apps such as Calm, Headspace, Breathe2Relax, Insight Timer, and Curable, none explicitly designed for headache. One clinician noted, however, that Insight Timer offers a mindfulness playlist explicitly designed for headache, which she recommends her patients download.

## Discussion

This generative research into current provider attitudes toward mobile health for headache yielded significant insight into current practices, provider goals, and impacts of mobile health on patient care. Interestingly, there was a considerable variation in providers' preferences and perceptions regarding electronic headache diary use. Most providers indicated that they had no preference between electronic or paper diary use, but that electronic tracking might allow for more detailed data. Many providers indicated they recommended mobile apps to their patients for headache tracking. Some providers indicated they only wanted limited data, such as headache frequency and duration.

In contrast, other providers expressed interest in additional data such as menstrual tracking and descriptions of symptoms. Most providers represented engagement with mobile health for headache in some capacity—most often through diary tracking and non-headache-specific behavioral interventions that could impact headache symptoms. Providers also disclosed their reservations regarding digital health care for a headache.

Providers noted that they recommend apps only to those patients who appeared sufficiently technologically capable of navigating apps. When assessing providers' perspectives on “capability,” most providers indicated that they took patient age into account. Specifically, if a patient was younger, they assumed they were more technologically savvy. Providers also indicated that they assessed for technological capability based on patient preference for paper or electronic headache tracking. However, it is unclear how all providers determine who is tech-savvy. Pre-conceived biases can influence who is perceived capable (14) and should be further assessed.

A recent review of commercially available headache apps identified Migraine Buddy (15), Migraine Monitor (16), and Migraine Coach (17) as having the best customizability, clinical accuracy, design efficacy, and user engagement (3). As the providers expressed contrasting views on what content they wished to obtain from patient diaries, varying preferences for app type may result. For instance, physicians with an interest in additional data might prefer Migraine Buddy, which contains a detailed clinical picture and includes features such as pain intensity and location, sleep tracking, as well as migraine triggers and allows users to download a report of their data (15). Alternately, Migraine Monitor allows physicians to access patient inputs and visualize patient data in real-time but has fewer diary features than Migraine Buddy (16). Physicians who wish to view limited data may prefer apps that are more succinct, like Migraine Monitor. Additionally, Migraine Coach was identified as an app that allows patients to track multiple headache-related symptoms but does not have an option for users to download their data (17). As some providers disclosed reservations about digital health care for headache, this app may be less popular due to its use of artificial intelligence chatting to answer users' questions about headache (17).

Providers frequently expressed a desire for apps providing behavioral intervention specifically for migraine. Reviews of the mobile health landscape for behavioral migraine therapy suggest a need for expert-reviewed apps and note that migraine-specific apps usually function as electronic headache diaries rather than offering behavioral interventions (18–20). In a recent review of headache management apps, 55 were identified, and only 18% included relaxation training such as diaphragmatic breathing or progressive muscle relaxation (20). Notably, apps with relaxation training did not have self-monitoring components for users to track their headache-related symptoms (20). Providers who wish to recommend an app with the inclusion of self-monitoring and behavioral intervention have limited options, resulting in a need to recommend more than one app.

Our team has studied the integration of behavioral intervention, specifically progressive muscle relaxation, into a headache diary through the app RELAXaHEAD in multiple settings (9–12).

Input from headache experts and providers serving various patients will be crucial to developing apps delivering behavioral interventions for migraine. However, this research indicates motivation on the part of providers to recommend such apps to patients. One of the barriers to developing apps with clinician input is app store publication requirements. If an app is considered a medical and/or diagnostic tool, the publication process is far more stringent than wellness apps. Many app developers sacrifice medical efficacy in favor of non-liability. These are crucial factors to remember when understanding why there are few commercially available apps clinicians trust in the marketplace.

### Comparison with prior work

Consistent with the “The 3 Fs:” Frequency of days with headache, Frequency of acute medication usage, and “Functional impairment” outlined in the previously conducted Modified Delphi study as the key categories of information clinicians seek out in patients' headache diaries, the providers interviewed in this study cited headache frequency, intensity, and medication use as the most clinically valuable outcomes or factors for patients to track (1) a minority of providers outlined an approach to headache diary use during appointments that involve the patient reporting concrete numbers for headache frequency, intensity, abortive/preventive medication use, and nothing more. These providers emphasized the relevance of headache diaries to patients, rather than providers, as educational tools for helping patients gain better insight into their condition. They also suggested that diaries may be most clinically useful when pinpointing a diagnosis early in treatment. Thus, the headache diary may serve separate, potentially overlapping purposes for providers and patients.

The same Modified Delphi study also characterized a proposed digital headache tracker as a shared decision-making tool for providers and patients (1). This aligns with reports from providers that use diary data to gain nuanced insight into patients' conditions (beyond what a patient may report verbally), such as the impact of headache on their daily functioning and realistic medication usage, which helps inform treatment plans and facilitates more productive office visits. More specifically, treatment response to abortive or preventative medications newly added to a patient's treatment plan. Patient symptom description and the timing of their headache may also be another helpful tool in confirming headache type and/or diagnosis.

Research suggests that diary applications are more accessible to patients than paper diaries and yield better adherence, which some providers represented was aligned with their experience—that patients were likely to report a headache that they currently had during the day on a phone they had with them than they were to return to a paper method after headache onset (21–23). A prospective observational study was recently conducted on a newly developed E-diary with an automated algorithm designed to distinguish between headache and migraine days based on patients' reported information; clinicians then determined headache or migraine days to inform patients' diagnoses (24). The E-diary was an effective strategy for mitigating the challenges posed by patients' limited ability to accurately remember and self-record their headache symptoms, which providers in this study reported as an imperative for asking patients to keep a headache diary. While many providers mentioned using diary data during visits to help inform treatment plans and assess the appropriateness of preventative therapies, others reported difficulty balancing the usefulness of this information with the burden of such detailed tracking on patients recording the data and on providers tasked with reviewing the data. Thus, further research into developing headache diaries must weigh the clinical benefits of accurately tracked diary data with the potential burden of more detailed E-diary tracking and reviewing such data for patients and providers, especially patients who report difficulty finding time to track their symptoms and providers who serve a high volume of patients.

### Strengths

This study has attained saturation with the 20 included participants despite the wide range of themes in the interview. In many cases, providers' attitudes toward mobile health were closely aligned with those of other providers, with minimal additional nuance based on personal experience or patients served. Minority opinions were often echoed thematically by a single provider, suggesting a good representation of thematic elements in the coding.

### Limitations

This study represents early qualitative research into perceptions and utilization of mobile health for headaches by current headache providers. While we reached data saturation, as with many qualitative studies, the sample size for this study is small as this is early qualitative research. Thus, it may not fully represent a wide population of clinicians. Possible self-selection bias occurred as providers may have opted into this research based on the current interest in and/or utilization of mobile health for headache. We had various experts review the questions before the study, but there is always the possibility of bias in the questioning. As mentioned above, providers made comments about not recommending apps to patients if they felt the patients needed to be tech-savvy. In future research, clinician age should be captured to identify if this may have influenced responses to interview questions.

Also, we did not ask providers whether they treat pediatrics and/or adults; thus, we need to find out if practice differs with these differences in age ranges. Pediatric providers have a different population of app users because the app user in pediatric settings is most likely to be a caregiver. As a result, the practice of app use may differ because the communication within the app is not coming directly from the patient. In future research, clarity on adult vs. pediatric clinicians is necessary.

## Conclusions

Future research should examine whether, once patients are designated part of a special population, it is useful to have additional features related to the population in the diary. Moreover, future research can expand to providers across professional settings (i.e., non-headache-specific settings). Further research into patient experience of providers' recommendations regarding mobile health for headache could expand understanding of the real-use implications of providers' beliefs regarding mobile health for headache. More specifically, future work might explore how to determine patients' readiness for mobile technology use.

## Data availability statement

The original contributions presented in the study are included in the article/Supplementary material, further inquiries can be directed to the corresponding author.

## Ethics statement

The studies involving humans were approved by NYU Langone Health Institutional Review Board. The studies were conducted in accordance with the local legislation and institutional requirements. The participants provided their written informed consent to participate in this study.

## Author contributions

MM: Conceptualization, Data curation, Formal analysis, Funding acquisition, Investigation, Methodology, Project administration, Supervision, Writing—original draft, Writing—review & editing. AG: Conceptualization, Data curation, Formal analysis, Investigation, Methodology, Project administration, Supervision, Writing—original draft, Writing—review & editing. NL: Conceptualization, Data curation, Formal analysis, Investigation, Methodology, Writing—original draft, Writing—review & editing. AK: Conceptualization, Data curation, Formal analysis, Investigation, Methodology, Writing—original draft, Writing—review & editing. IS: Conceptualization, Data curation, Formal analysis, Investigation, Methodology, Project administration, Validation, Writing—original draft, Writing—review & editing.

## Appendix

**Table A1:** Semi-structured interview questions.

**Category**	**Question**
Current practices	- Do you recommend that your headache patients track their headaches or utilize a headache diary? - If so, how? Do you recommend or suggest any specific methodologies for tracking? Why? - Do you recommend that your headache patients use any apps for learning about and practicing behavioral therapy for headache? - If so, how? Do you recommend or suggest any specific behavioral tools for this? Why?
Impact of diaries	- What is the most important outcome of your patients utilizing a headache diary? - How is information from these diaries utilized during appointments?
Interest in digital	- Have you ever recommended a digital program or app for headache? - If so, which one(s)? What have you liked/disliked about them? What determines to whom you recommend a digital program or all for headache? What determines which patients you recommend a digital option to vs. paper and pencil for tracking? - If not, would you consider recommending that your patients utilize an electronic headache diary? Why or why not?
Workflow	- If you could view a patient's headache diary information outside of an appointment, would you be interested in this kind of remote monitoring? Why or why not? - How do you imagine this kind of electronic headache diary and data monitoring would integrate into a headache visit with a provider?
The benefit of digital	- How would you imagine this kind of data monitoring to affect your appointment visits with patients? What, if anything, would it change? What might be the benefits? - How might a provider, if at all, leverage this data in the most helpful way?
Concerns re digital	- What potential issues can you imagine with this kind of remote monitoring and electronic headache diary usage?
Considerations beyond patient-provider interaction (e.g., institutional, liability)	- Taking the quality of patient care and any time considerations for you out of it, what other considerations might impact your perspective on these technologies? - How would you imagine this kind of strategy interacting with an institution's IT/technology needs and capabilities, specifically around integration with the EMR or liability? - What are your thoughts about the level of support and/or pushback you might get from your institution for integrating digital monitoring into your workflow?
