# Supplementary material for: Headache providers' perspectives of headache diaries in the era of increasing technology use: a qualitative study
Source: Front Neurol. 2024 Jan 23;14:1270555. doi: 10.3389/fneur.2023.1270555 (PMC10844531; doi:10.3389/fneur.2023.1270555)
Supplement: Supplementary file 1 [file Table_1.docx]

**Supplemental Table 1.** Headache Diary Themes and Subthemes

| **Theme** | **Quotes** |
| --- | --- |
| **Providers were generally agnostic regarding the headache tracking method** | "Migraine Mentor collects frequency severity, duration, and disability. It also looks at activity, sleep, eating schedule. Subjectively, suspected triggers, environmental changes like barometric pressure change, traffic patterns, things like that, and then it creates a spider diagram." (Clinician 5)  “So, I don't use zero to ten, I use green, yellow or none and then green, yellow, red. So, I ask them to write down those colors. Migraine buddy, for example, does not do it that way so it's a little confusing to patients because then they're switching back to the number thing, but I try and get them to do that and then what medicine they use and the duration of the headache.” (Clinician 10)  “I usually have them keep a notes section in their phones or I have them do the stoplight journal, which is the three different colors of the red, green, and yellow days, so they might just write the word of the color next to it in their phone, and then the third option for anyone who’s a little more tech-savvy would be the Migraine Buddy app. So those are kind of the three choices, and then I decide based on what kind of data I’m looking for, which would I recommend for that patient.” (Clinician 13)  “It’s just I haven’t seen a successful electronic diary that’s above a 10-20% sustained use rate that would make it worth my patients’ time say they should dedicate their time to that. I would like to find one, but it just doesn’t exist." (Clinician 3) |
| **Providers had concerns regarding the accessibility of headache trackers** | “I mean usually if they’re older, then the paper and pencil versus younger would do apps. It's just whatever is simpler.” (Clinician 4)  “Multiple reasons. One might be ease of which or how fast they are with technology. And that has many factors: age, personal preference, lifestyle, access to resources.” (Clinician 12)  “I think a big limitation is that we don’t have any contact to help us, you know? Because when you have technology, it’s like, I don’t think it should be on the doctor to try to do IT management for patients, but there’s also no contact, staff is not helping and then it’s kind of like the patient asking me to solve the technological problems of logging in the app or something, and I don’t know, I really don’t know how to manage my own phone myself.” (Clinician 16) |
| **Providers noted benefits to integrating headache tracking data into the EMR** | “So, a patient will upload it to our MyChart system that is the patient portal where they can message a provider. They will upload it as like a PDF attachment or I'll ask them to just have their headache calendar with them during the visit and then they can report to me the numbers.” (Clinician 17)  “So, that’s one of the things that we tried earlier, or a year ago, was we had a system that interfaced with our health record, and so, you could have the option as the provider to preview it, which I’m horrible at, looking at before the office visit.” (Clinician 1)  “So, I think it is a great tool; if we can incorporate it into our electronic medical system that would be even better. And I am excited to see how other institutions are doing it if they start implementing it because it would make a big, big difference in our practice." (Clinician 2)  “Yeah, so all of our patients have been asked to use [Nerivio], but then part of the issue is that it’s not linked to Epic, so basically, then patients have to take screenshots and send us a message with the headache diary.” (Clinician 16) |
| **Providers had mixed opinions regarding the utility and interpretation of the data, specifically regarding data accuracy and efficiency** | “I just think back to the one app that we used, one thing that was difficult to track was, duration of a headache cycle, so you could see numbers of days, but it’s hard to catch that it was one migraine that lasted three days, there were some little bit of time constraint, or limitations on some of the data that was recorded, but readability was fine, cause you can do it graph form, numbers of days, summary tables, in the apps that I’ve seen.” (Clinician 1)  “I have, like a kind of a running chart in my notes where I kind of keep track of their headache frequency and intensity at each visit. So, we can kind of, like, as I enter that data, we get a sense of where they're going over time, and then that plays a big influence on where we want to go next with sort of our treatment modalities.” (Clinician 7)  “So, it's easier to read and to see how the patient is doing, I think. It's faster, I think, in a way. Once you have the digital app, like, you have all the information I'm looking for.” (Clinician 9)  “No, I don’t even necessarily care to look at it as long as they [patients] tell me the number of headaches a month and try to extract triggers for themselves.” (Clinician 14) |
| **Providers generally felt that headache tracking lends itself to more collaborative plan management** | “I think they would like the aspect of having that control, and yeah and having control over their decisions in their healthcare because they are utilizing the app that we are also using. So, independence and control from their end, but also maybe confusion and expectation that technology could be replacing human interaction, which is how I don’t see it, but some people may.” (Clinician 2)  “They're doing something objectively so that they can start to get a grip on [their headaches]. And it's not just me telling them use this preventive or do this, do that. I feel like it'll help them to develop their locus of control.” (Clinician 6)  “So, I think there is this outcome, which is maybe related to self-efficacy but not only—self-understanding, self-acknowledgement, understanding their behavior and their condition’s behavior.” (Clinician 8)  “I see a lot of patients won't do it is because they don't want to be reminded every day. And then all patients might start out using it but then as time goes on they will stop using it because most of the time it's a good thing meaning that, you know like, my headaches have become so infrequent that I don’t need to track them or, I slept like every day, I'm so sick of inputting this, I have the same pain every day, so sometimes that will deter them from using it if they're not seeing changes in their headaches.” (Clinician 11) |
| **Providers recommend behavioral health apps for patients** | “We recommend free apps for relaxation and breathing, sleeping—Calm, Headspace. And the behavioral therapy and behavioral therapy for insomnia as far as apps I’d like to recommend, I just haven’t looked deeply into it.” (Clinician 15)  “We tell them you know those are things you can look up - such as mindfulness and meditation, things like that - but there's no specific apps that we recommend.” (Clinician 18)  “Headspace and Calm, I don’t even remember where I first heard about them, could be from patients as well, but I personally checked out Headspace, I thought I liked it, I think it’s nice. Calm, I don’t know if I, myself, tried but lots of people have. And This Way Up, a colleague recommended it specifically for people for CBT. That one is, I think, a little bit pricier, so for people that I think would specifically benefit from CBT, that’s the one I recommend.” (Clinician 19) |
